# Organization of Medical Society of Waupaca County, Wisconsin

**Published:** 1858-02

**Authors:** Isaac E. Thayer, Geo. H. Colkins

**Affiliations:** President; Rec. Sec.


### ORGANIZATION OF MEDICAL SOCIETY OF WAUPACA COUNTY, WISCONSIN.

In accordance with previous notice, the physicians of the County of Waupaca convened at the Court House, in the village of Waupaca, on Wednesday, September 23d, 1857, and proceeded to organize a County Medical Society, by electing the following officers for the ensuing year:

President, . . . . . . . Isaac E. Thayer, M. D.
Vice-President, . . . . B. W. Foss, M. D.
Recording Secretary, . . Geo. H. Colkins, M. D.
Corresponding Secretary, . Parley Dickinson, M. D.
Treasurer, . . . . . . . Geo. R. Taylor, M. D.
Censors—Drs. L. B. Brainard, C. Linde, and T. Lawyer.

Drs. Foss, Taylor and Colkins were appointed a committee to draft constitution and by-laws, to be presented at next meeting.

Dr. C. Linde was chosen delegate to the State Medical Society.

On examination, the Board of Censors reported favorably to the admission of John S. Pashley and J. Warren Perry, and on motion they were admitted as members.

The local papers and *Chicago Medical Journal* were requested to publish the above proceedings.

The society then adjourned to meet at Weyanwega on the first Thursday in April, 1858.

ISAAC E. THAYER, *President*.

GEO. H. COLKINS, *Rec. Sec.*

